# Lectures on Massage and Electricity in the Treatment of Disease

**Published:** 1890-12

**Authors:** 


Lectures on Massage and Electricity in the Treatment of Disease (Masso-Electro-therapeutics). By Thomas Stretch Dowse, M. D., Fellow of the College of Physicians of Edinburg; formerly Physician Superintendent of the Central London Sick Asylum; Associate Member of the Neurological Society of New York, etc. Royal octavo, cloth, pp. 373. New York: E. B. Treat & Co., 5 Cooper Union. London; Hamilton, Adams & Company. 1890. Price, $2.75.

This book comprises a series of fifteen lectures.

Those on Massage are good, and will give the reader a very fair idea of the methods used in applying the treatment. This is made more easy by the aid of numerous illustrations. The lectures are strengthened by lengthy quotations from other authors, notably, Charcot, Weir Mitchell, and Graham. Like most writers on this subject, the author is enthusiastic, and leaves the impression on the reader that massage will accomplish more than we have found in our experience. He recommends it also as a treatment for housemaid's knee. This is recommending a slow and tedious process in place of the more rapid and more certain surgical treatment.

If advocates of massage would recommend it for use where it is the best treatment, we believe it would come far more rapidly into general use.

The lectures on electricity are too curtailed to do much more than give a general idea of electro-therapeutics.

The volume is well published, and is remarkably free from typographical errors. J. W. P.

